# YKL-40 (Chitinase 3-like I) is expressed in a subset of astrocytes in Alzheimer’s disease and other tauopathies

**DOI:** 10.1186/s12974-017-0893-7

**Published:** 2017-06-09

**Authors:** Marta Querol-Vilaseca, Martí Colom-Cadena, Jordi Pegueroles, Carla San Martín-Paniello, Jordi Clarimon, Olivia Belbin, Juan Fortea, Alberto Lleó

**Affiliations:** 1 Memory Unit, Department of Neurology, Institut d’Investigacions Biomèdiques Sant Pau - Hospital de la Santa Creu i Sant Pau, Universitat Autònoma de Barcelona, Sant Antoni M. Claret 167, 08025 Barcelona, Spain; 2 Centro de Investigación Biomédica en Red en Enfermedades Neurodegenerativas (CIBERNED), Madrid, Spain

**Keywords:** YKL-40, Alzheimer’s disease, Tauopathies, Tau, Astrocytes, Neuroinflammation

## Abstract

**Background:**

The innate immune system is known to be involved early in the pathogenesis of Alzheimer’s disease (AD) and other neurodegenerative disorders. The inflammatory response in the central nervous system can be measured postmortem or through a series of inflammatory mediator surrogates. YKL-40 (also named Chitinase-3-like I) has been frequently investigated in body fluids as a surrogate marker of neuroinflammation in AD and other neurological disorders. However, the expression pattern of YKL-40 in the human brain with neurodegenerative pathology remains poorly investigated. Our aim was to study the cellular expression pattern of YKL-40 in the brain of patients with clinical and neuropathological criteria for AD (*n* = 11); three non-AD tauopathies: Pick’s disease (PiD; *n* = 8), corticobasal degeneration (CBD; *n* = 8) and progressive supranuclear palsy (PSP; *n* = 9) and a group of neurologically healthy controls (*n* = 6).

**Methods:**

Semiquantitative neuropathological evaluation and quantitative confocal triple immunofluorescence studies were performed. An in-house algorithm was used to detect and quantify pathology burden of random regions of interest on a full tissue-section scan. Kruskal-Wallis and Dunn’s multiple comparison tests were performed for colocalization and quantification analyses.

**Results:**

We found that brain YKL-40 immunoreactivity was observed in a subset of astrocytes in all four diseases and in controls. There was a strong colocalization between YKL-40 and the astroglial marker GFAP but not with neuronal nor microglial markers. Intriguingly, YKL-40-positive astrocytes were tau-negative in PSP, CBD and PiD. The number of YKL-40-positive astrocytes was increased in tauopathies compared with that in controls. A positive correlation was found between YKL-40 and tau immunoreactivities.

**Conclusions:**

This study confirms that YKL-40 is expressed by a subset of astrocytes in AD and other tauopathies. YKL-40 expression is elevated in several neurodegenerative conditions and correlates with tau pathology.

**Electronic supplementary material:**

The online version of this article (doi:10.1186/s12974-017-0893-7) contains supplementary material, which is available to authorized users.

## Background

There is growing evidence that the immune system is involved early in the pathogenesis of Alzheimer’s disease (AD) and in other neurodegenerative diseases [1, 2]. The activation of the immune system in AD (often referred to as “neuroinflammation”) is known to be present at all stages of AD and is believed to play an active role in the disease process. The activation of microglia and astrocytes as a reaction to ongoing deposition of Aβ triggers the production of several proinflammatory signal molecules including cytokines, chemokines, complement molecules, growth factors and cell adhesion molecules [3]. The recent association of gene encoding inflammatory proteins, such as TREM2 and CD33 with AD [4–6], has further supported the role of the innate immune response in the aetiology and progression of AD. In addition, the increased feasibility of measuring a wide range of inflammatory molecules in biofluids from patients at different AD stages has expanded our understanding about the type of immune responses observed in neurodegenerative diseases. A variety of cytokines, chemokines and other inflammatory mediators are increased in cerebrospinal fluid (CSF) or in plasma in AD [1]. One of the proteins that has been frequently measured in body fluids as a surrogate marker of neuroinflammation in AD and other neurological disorders is YKL-40 (also named Chitinase 3-like I) [7, 8]. It has been described that YKL-40 participates in connective tissue cell growth, endothelial cell migration and inhibition of mammary epithelial cell differentiation and promotes tumour angiogenesis [9]. However, its biological and physiological functions in the central nervous system remain unclear. YKL-40 is increased in the CSF of multiple sclerosis (MS) patients, and YKL-40 levels correlate well with disease progression [10]. In previous studies, we and others [11, 12] found elevated YKL-40 levels in the CSF of AD patients. Interestingly, increased levels of YKL-40 were found in the preclinical stages of AD [13, 14] indicating that the immune system activation occurs early in the disease. These studies have also shown that CSF levels of YKL-40 and tau strongly correlate [11, 12]. Additional studies have indicated that YKL-40 is also elevated in the CSF of patients with other tauopathies, such as frontotemporal dementia (FTD), corticobasal degeneration (CBD) and progressive supranuclear palsy (PSP) [11, 13, 15, 16].

Despite the wide use of YKL-40 as a biochemical marker in neurodegenerative diseases, its distribution and pattern of expression in the human brain remains unclear. A recent study found that expression levels of chitinase genes in the brain regions of late onset AD (LOAD) patients are increased compared with healthy controls [17], but the cellular source of expression of these proteins remains uncertain. Some studies have suggested that YKL-40 is expressed in astrocytes in a variety of acute neuroinflammatory conditions, such as traumatic brain injury or multiple sclerosis [18, 19]. Other studies, however, support the idea that YKL-40 is also expressed in macrophage/microglia cell types in these conditions [19, 20]. In AD, studies show a variable pattern of YKL-40 expression that includes astrocytes, microglia or, on rare occasions, neurons [12, 21, 22]. The aim of the present work was to determine the cellular pattern of YKL-40 expression in the human brain tissue in AD and other tauopathies. We also investigated the relationship between YKL-40 expression and tau aggregates in these disorders.

## Methods

### Standard protocol approval and patient consent

A written informed consent was given by all donors and/or next of kin for the use of brain tissue for research. This study was approved by the local ethics committee at Hospital de Sant Pau, Barcelona, Spain.

### Human brain samples

Human brain samples were provided by the Neurological Tissue Bank (NTB) of the Biobanc-Hospital Clínic-IDIBAPS and processed as previously described [23] and as internationally recommended [24]. The initial study group consisted of 40 patients who met clinical and neuropathological criteria for AD (*n* = 11), PSP (*n* = 10), CBD (*n* = 9), Pick’s disease (PiD; *n* = 10) and a group of healthy controls (*n* = 7).

### Neuropathologic assessment

We assessed formalin-fixed and paraffin-embedded tissue blocks from frontal cortex Brodmann areas 8/9. Immunohistochemistry was performed on 5-μm-thick sections on an automated stainer (DAKO Autostainer Plus; DAKO, Glostrup, Denmark) using the following primary antibodies: anti-amyloid β (clone 6F/3D, dilution 1:400; DAKO) and anti-phosphorylated tau (clone AT8, dilution 1:2000; Thermo Scientific, Waltham, MA). Reaction was visualized by the EnVision+ system peroxidase procedure (DAKO).

Immunoreactive structures of AT8 (NFTs, NTs, pretangles, dystrophic neurites, balloon cells, Pick bodies, ramified astrocytes, astrocytic plaques, tufted astrocytes and coiled bodies) and β-amyloid (mature, primitive and diffuse plaques) were systematically assessed in all cases. Neurofibrillary pathology was staged according to Braak criteria [25, 26]. β-amyloid phases were evaluated according to Thal criteria [27]. The National Institute on Aging-Alzheimer’s Association Guidelines for neuropathologic assessment of AD was also applied [24]. Healthy controls without tau or amyloid pathology were included. Neuropathologic evaluation was carried out by three investigators on a multiheaded microscope.

### Immunohistochemistry (IHC) and immunofluorescence (IF)

Formalin-fixed and paraffin-embedded brain sections of frontal cortex were dewaxed and pretreated with Tris/EDTA buffer pH 9 at high temperature. The following primary antibodies were incubated overnight at 4 °C: polyclonal goat anti-YKL-40 (R&D Systems, AF2599, dilution 1:200), rabbit anti-GFAP (Sigma, G9269, dilution 1:500), phosphorylated tau clone AT8 (Thermo Scientific, MN1020, dilution 1:1000), monoclonal mouse anti-MAP2 (Sigma, M4403, dilution 1:500) and rabbit anti-Iba-1 (Wako Chemicals, 019-19741, 1:500). For IHC, the endogenous peroxidase activity was blocked, sections were HRP-labelled (Dako, Glostrup, Denmark, dilution 1:200) and the reaction was visualized by the EnVision+ system peroxidase procedure (DAKO, Glostrup, Denmark). For IF, sections were incubated for 1 h with Alexa Fluor 488, 555 or 647 (Invitrogen, Carlsbad, CA, USA, dilution 1:1000) secondary antibodies and stained with Sudan black B (Merck, Whitehouse Station, NJ, USA) to mask tissue autofluorescence. Nuclei were stained with Hoechst 33258 (Life Technologies, Carlsbad, CA, USA, dilution 1:1000), and coverslips were added with Immu-Mount (Fisher Scientific, Rockford, USA) mounting medium.

### Image acquisition and analysis

Fluorescence images were acquired with a Leica inverted fluorescent confocal microscope (Leica TCD SP5-AOBS, Wetzlar, Germany) with a ×40 1.4 NA oil objective. Alexa Fluor 488, 555 and 647 were sequentially excited with 488-, 561- and 633-nm laser lines and captured with a spectral window of 498 to 530, 571 to 620 and 645 to 720 nm, respectively. A pulsed 405-nm laser was used for Hoechst visualization capturing images in a spectral range of 415 to 475 nm. Sections without antibodies or with secondary antibodies only were imaged to ensure specific and independent fluorophore visualization. For each case, at least 10 images per area were acquired. Pictures were taken in 4–5 z planes with a 0.7-μm pinhole. Maximal intensity projection of each type of aggregate was used for figure visualization.

For colocalization analyses, images were acquired avoiding saturated pixels. Saturation was only minimally applied for presentation purposes in the figure. Protein colocalization was evaluated using FIJI imaging software [28]. All images were analyzed following the same semi-automated in-house algorithm. Briefly, for each channel, the lowest intensity signals within a z-stack were removed to minimize background. An automated threshold was then estimated to create binary images. To quantify the overlap between proteins, Manders’ colocalization coefficient was calculated for each channel [29, 30].

For quantification analysis of IHC stains, full-section scans were obtained with Pannoramic MIDI II (3DHistech, Budapest, Hungary). Cortical grey matter of each case was delimited blinded to clinical phenotypes. An in-house computer-based algorithm was developed to quantify tau pathology burden and GFAP immunoreactivity with MATLAB R2015b software (The MathWorks, Inc., Natick, MA, USA) (Additional file 1: Figure S1). Briefly, the algorithm allows defining random regions of interest (ROIs) on a full-section scan, to compute densities of protein expression and to quantify the number of immunoreactive objects. This procedure was adapted from a published digital image analysis [31]. An outlier test was performed, and cases reported as statistical outliers were removed from the analysis. Five cases were excluded such that the final sample included the following cases: 11 AD, 9 PSP, 8 CBD, 8 PiD and 6 healthy controls. All quantitative analyses performed with the developed semi-automated method were manually validated in a subset of images. YKL-40-positive objects were manually validated by two investigators in order to ensure the correct identification of immunoreactivity patterns by the automated algorithm. Both investigators were blinded to clinical phenotypes.

### Statistical analysis

Kruskal-Wallis and Dunn’s multiple comparison tests were performed for colocalization and quantification analyses. Correlation between proteins was measured by the Spearman coefficient. Statistical significance was set at 5% (*α* = 0.05). All data were analyzed using the GraphPad Prism 6.0 software (GraphPad Software, Inc., CA, USA).

## Results

### YKL-40 is expressed by astrocytes in human brain tissue

**Fig. 1:**
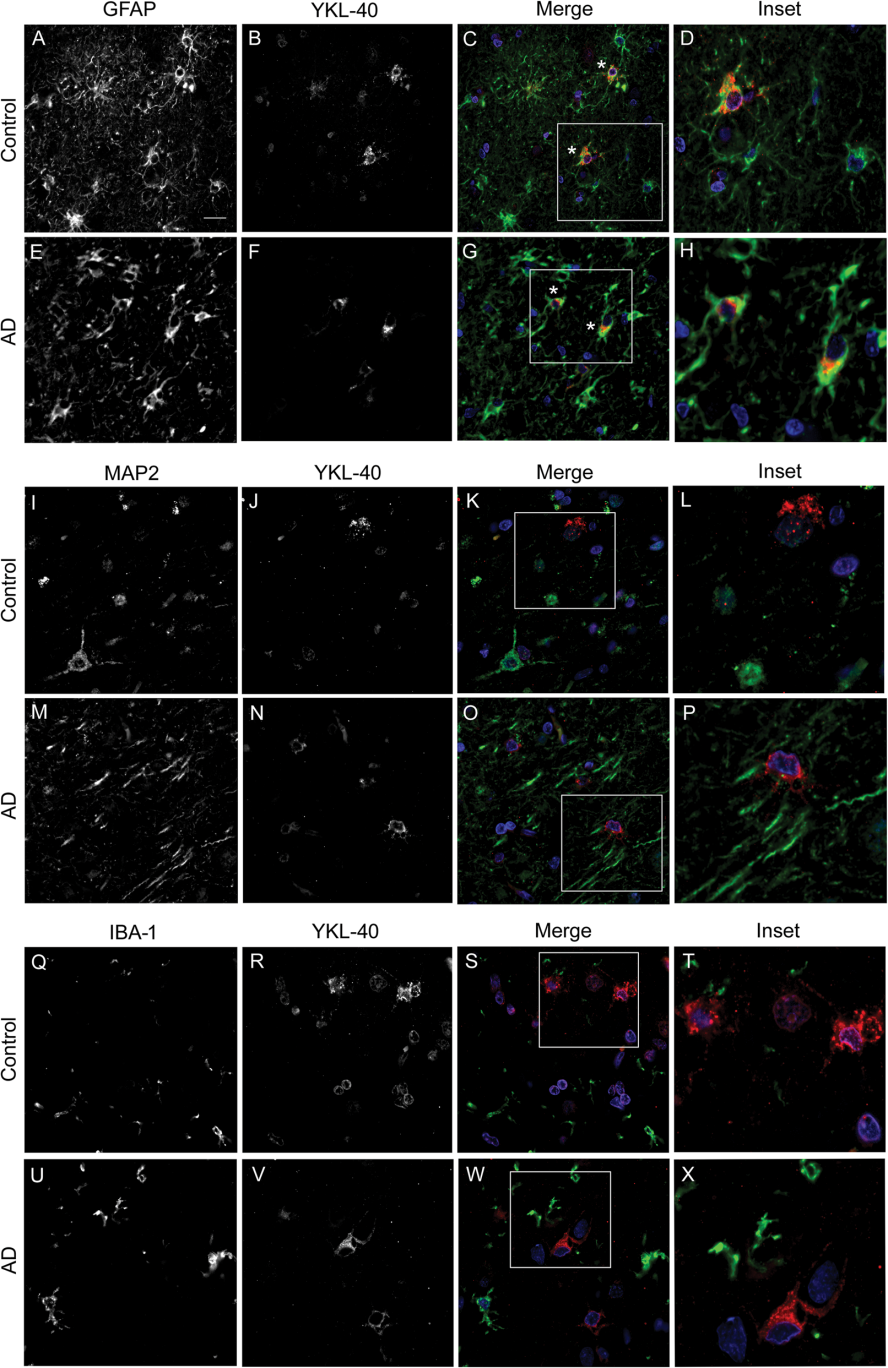
YKL-40 expression pattern in human brain tissue from an AD patient and a healthy control. Representative images of double immunofluorescence performed with YKL-40 (*red*) and three different cellular markers, GFAP (astroglial, *green*), MAP2 (neuronal, *green*) and IBA-1 (microglial, *green*). Nuclei are marked in *blue*. **a**–**h** YKL-40 immunoreactivity was detected in the cytoplasm of GFAP+ cells (*asterisk*), indicating an astroglial origin. **i**–**p** No colocalization was observed between YKL-40 and the neuronal marker, MAP2 or (**q**–**x**) with the microglial marker, IBA-1. Scale bar = 20 μm

We first examined the cellular expression of YKL-40 in human brain tissue from an AD patient and a healthy control. Using a double immunofluorescence technique, we investigated the colocalization between YKL-40 and three different markers: MAP2 as a neuronal marker, GFAP as an astroglial marker and IBA-1 as a microglial marker (Fig. 1). In both the AD and control brains, we found that YKL-40 colocalized in some cells labelled with the astrocytic marker GFAP, with a perinuclear cytoplasmic pattern extending to some proximal astroglial processes (Fig. 1a–h). Conversely, there was no colocalization between YKL-40 and the neuronal marker, MAP2, or with the microglia marker, IBA-1 (Fig. 1i–x). These findings indicate that YKL-40 is expressed by astrocytes in the frontal cortex of healthy and diseased human brain tissue.

### YKL-40 is expressed in a subset of astrocytes in AD and other non-AD tauopathies

We next investigated whether the pattern of YKL-40 was similar between AD and other neurodegenerative diseases. YKL-40 expression pattern was examined in the frontal cortex from patients with different forms of frontotemporal lobar degeneration (FTLD) such as PiD, CBD and PSP. Since tau is known to aggregate in astrocytes in these subtypes of FTLD [32], we explored the relationship between YKL-40 expression and the different forms of tau aggregates in these conditions.

**Fig. 2:**
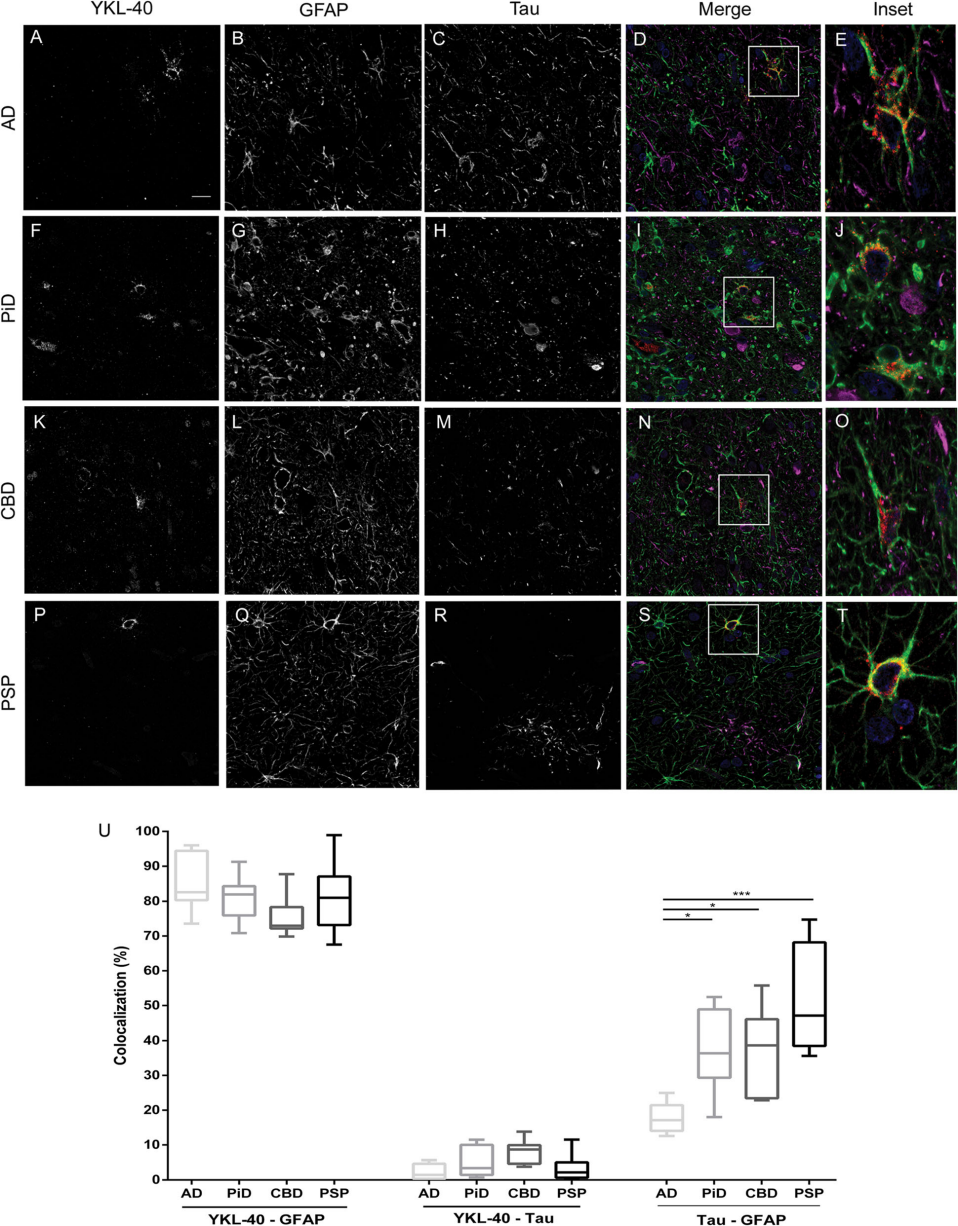
YKL-40 immunoreactivity pattern and colocalization analyses in different tauopathies. Representative images of the triple immunofluorescence studies performed with YKL-40 (*red*), GFAP (*green*) and tau (*magenta*) antibodies. Nuclei are marked in *blue*. All four tauopathies investigated, **a**–**e** AD, **f**–**j** PiD, **k**–**o** CBD and **p**–**t** PSP, showed a cytoplasmic astroglial expression pattern of YKL-40. Scale bar = 20 μm. **u** Colocalization analysis confirmed that approximately 80% of YKL-40 colocalized with GFAP in all tauopathies. No colocalization was detected between YKL-40 and tau in any condition. As expected, non-AD tauopathies showed an overlap between tau and GFAP. ***p < 0.001; *p < 0.05

We performed triple immunofluorescence studies with antibodies against YKL-40, GFAP and tau in different cases of AD and non-AD tauopathies (*n* = 40) (Fig. 2). We confirmed that 75–85% of YKL-40 immunoreactivity colocalized with GFAP indicating an astroglial origin in cases with AD, PiD, CBD and PSP (Fig. 2u). The immunoreactivity of YKL-40 was mainly cytoplasmic while GFAP immunoreactivity extended distally to astrocytic processes and plasma membrane, which explains the lack of complete overlap. No differences in the expression pattern of YKL-40 between the AD and FTLD cases were found (all *p* > 0.05). Interestingly, in PSP, CBD and PiD, where tau-positive astrocytes are commonly found, most YKL-40-positive astrocytes were tau negative and vice versa. Accordingly, colocalization between YKL-40 and tau was negligible (<7%). On the other hand, as previously described [32], substantial overlap between tau and GFAP immunoreactivity was found in PiD (~37%), CBD (~37%) and PSP (~51%), whereas no overlap was found in AD. The distribution of YKL-40-positive astrocytes was mainly isolated but occasionally found surrounding blood vessels. These data support the idea that the immunoreactivity patterns of astroglial YKL-40 and tau are spatially distinct in non-AD tauopathies.

### Relationship between YKL-40 expression, tau pathology and astrogliosis

**Fig. 3:**
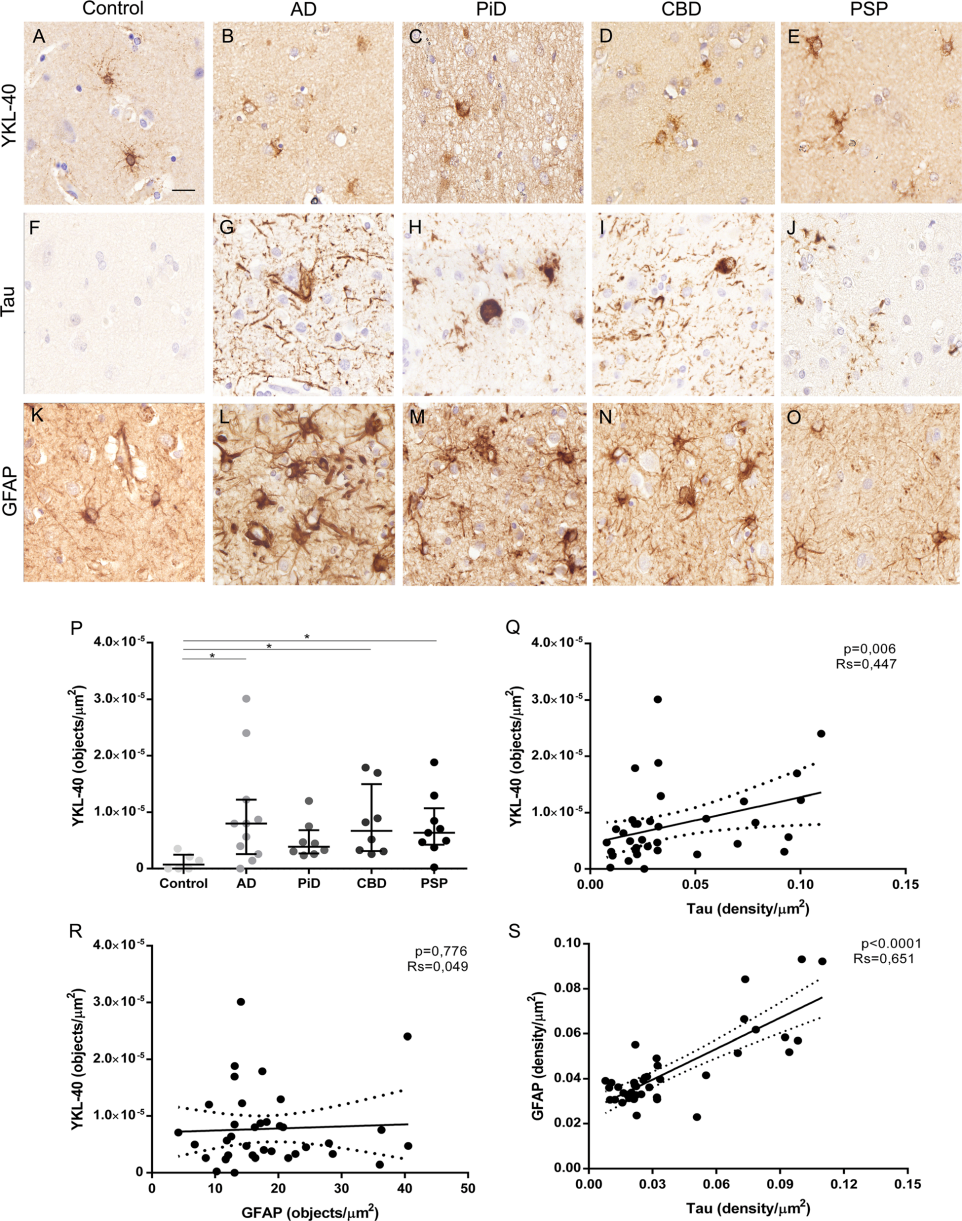
Quantification of YKL-40, tau pathology burden and astrogliosis in different tauopathies. **a**–**o** Representative images of YKL-40, tau and GFAP immunoreactivity of controls and the four tauopathies under study. **a**–**e** YKL-40 expression pattern. **f**–**j** Main tau deposits and aggregates of each condition. **k**–**o** Astrogliosis among the tauopathies and controls. Scale bar = 20 μm. **p** YKL-40 immunoreactivity (objects/μm^2^) measured in all conditions. **q**–**s** Correlation between different markers. Levels of YKL-40 and GFAP correlated positively with tau aggregation. *Solid lines* indicate the linear regression, and *dotted lines* indicate 95% CI. *RS*, Spearman rho coefficient.

We next investigated the differences in total YKL-40 immunoreactivity between the neurodegenerative conditions and healthy control brains. Immunohistochemistry for YKL-40, GFAP and tau was performed on three consecutive sections from the frontal cortex from each case, respectively. Full-section scans of all samples were obtained, and quantification of tau, YKL-40 and GFAP was performed. Representative images of YKL-40, tau and GFAP immunoreactivity are shown in Fig. 3(a–o). A similar YKL-40 expression pattern was observed across all neurodegenerative conditions although expected differences in tau deposits were detected between conditions.

We next applied our in-house semi-automated algorithm to quantify total immunoreactivity (density/μm^2^) and number of objects of Tau and GFAP markers (Additional file 1: Figure S1). YKL-40 levels were increased in all neurodegenerative diseases (except PiD) compared with controls (*p* < 0.05). No differences were observed between the different tauopathies (Fig. 3p). Less than 10% of GFAP-positive astrocytes expressed YKL-40 (10% in PSP, 7% in CBD, 5% in AD, 4% in PiD and 1% in healthy controls).

We next examined the correlation between YKL-40, tau and GFAP markers. We found a positive correlation between tau pathology burden scores and YKL-40 (*n* = 36; *r* = 0.447; *p* = 0.006) (Fig. 3q) and between GFAP and tau pathology (*n* = 36; *r* = 0.651; *p* < 0.0001) (Fig. 3s). However, we did not detect a correlation between YKL-40 and GFAP (*n* = 36; *p* = 0.776) scores (Fig. 3r).

## Discussion

In the present study, we found that YKL-40 is expressed by astrocytes in human brain tissue in healthy controls and in different neurodegenerative diseases. The immunoreactivity pattern of YKL-40 was mainly cytoplasmic extending to proximal astrocytic processes. We also observed that this protein is expressed in a subset of astrocytes (<10%) that do not contain tau aggregates in non-AD tauopathies.

To date, the pattern of expression of YKL-40 in the central nervous system has remained controversial and has not been fully elucidated. Some studies have described that YKL-40 is expressed in microglia [33, 34] while others have found it in astrocytes [12, 35]. These discrepancies may be because YKL-40 expression seems to vary depending on the disease and the severity of the neuroinflammatory response [19, 35]. It has been shown that in multiple sclerosis, YKL-40 is expressed by macrophages/microglial cells (CD68+) in low and high inflammatory activity lesions [19]. However, YKL-40 expression was also expressed in the cytoplasm of astrocytes (GFAP+) in high inflammatory activity lesions [19]. Another study reported that in human brain infarction, YKL-40 astrocytic expression depends on the stage of underlying inflammation, increasing during the acute inflammation phase and diminishing as the inflammation resolves [35]. Moreover, it has been shown by others that YKL-40 is also expressed by peripheral cells including chondrocytes [36], synoviocytes [37], vascular smooth muscle cells [38], macrophages [39] and neutrophils [40]. For example, it has been reported that expression of YKL-40 in breast cancer tissue correlates with tumour grade [41] and that YKL-40 macrophage expression is upregulated in patients with chronic obstructive pulmonary disease and correlates with its severity [42]. In vitro studies have shown a dramatically increase of YKL-40 expression during astrocyte differentiation [43]. Other studies based on immunohistochemical techniques suggested that YKL-40 together with SSEA-4 marker expression represent an unexplored astrogenic lineage [44]. Here, we have confirmed that YKL-40 shows an astroglial cytoplasmic immunoreactivity pattern in postmortem human frontal cortex from healthy controls and AD patients. We did not detect YKL-40 expression in microglia or in neurons.

We also found YKL-40 in the cytoplasm of astrocytes in non-AD tauopathies, including PiD, CBD and PSP. Although YKL-40 was typically found in isolated astrocytes, we occasionally observed focal astrocytic YKL-40 immunoreactivity around blood vessels. Previous studies have suggested that YKL-40 transcription is induced in astrocytes by proinflammatory factors released from macrophages [21]. One possible explanation for this observation is that perivascular macrophages may induce YKL-40 expression in astrocytes that are in close proximity. The colocalization between YKL-40 and GFAP was around 80% indicating that GFAP immunoreactivity, typically extending towards the astrocyte membrane and processes, surrounds that of YKL-40. Interestingly, YKL-40 immmunoreactivity was independent of tau, indicating that in non-AD tauopathies, YKL-40 is expressed in a tau-negative subset of astrocytes. Whether YKL-40 could be a compensatory response to inhibit tau aggregation or, on the contrary, represents an initial event that facilities tau deposition requires further investigation. As expected, colocalization between tau and GFAP was found in non-AD tauopathies reflecting the distinctive glial tau aggregation in these disorders [32]. The 15% overlap observed in AD may be explained by neuropil threads crossing astrocyte processes in close proximity that are measured as colocalization due to the resolution limits.

Recent studies have demonstrated that YKL-40 levels are increased in the CSF of patients with AD and FTD compared with those in the healthy controls [11–16, 45, 46]. In addition, a positive correlation between YKL-40, total tau and p-tau has been reported in CSF, suggesting that inflammation and tau-associated neurodegeneration are related pathophysiological processes. In agreement, animal models and co-culture studies have shown that activated glia-induced neuronal tau phosphorylation and aggregation [47]. In postmortem brains, our semi-automated method revealed that total YKL-40 levels were statistically increased in all tauopathies (except PiD) compared with healthy controls. These results are in agreement with studies that investigated YKL-40 levels in CSF of AD and FTD patients [11–16, 45, 46]. It is important to note that only a limited proportion of astrocytes (always less than 10%) expressed YKL-40 in human frontal cortex. Interestingly, we found a positive correlation between YKL-40 and tau pathology burden (*r* = 0.447) suggesting that inflammation and neurodegeneration may be closely related processes in humans. The lack of correlation between YKL-40 and GFAP together with the positive correlation between tau and GFAP (*r* = 0.651) in our study also may indicate that YKL-40 expression is independent of astrocyte activation in neurodegenerative disease.

## Conclusions

In conclusion, this is, to our knowledge, the first detailed neuropathologic characterization of YKL-40 expression in human brain tissue. Moreover, the study includes tissue samples from healthy controls and four neurodegenerative diseases. Combining confocal microscopy and the application of a semi-automated method to quantify pathology burden, we have shown that the immunoreactivity pattern of YKL-40 in AD and other tauopathies is astroglial. YKL-40 is expressed by a subset of astrocytes that do not contain tau aggregates in non-AD tauopathies. Finally, we have found that YKL-40 inflammatory marker is associated with tau pathology in neurodegenerative diseases that accumulate tau.

## Additional file

Additional file 1:
**Figure S1.** Semi-automated method for pathological burden quantification. For all conditions tau and GFAP were assessed using a randomized computer-based quantification of patterns and severity in immunohistochemical stains. Cortical grey matter of each case was delimited blinded to clinical phenotypes (A). We developed an in-house algorithm that allows defining randomized regions of interest (ROIs) on a full-section scan (B–C), to compute density of protein expression (D) and to quantify the number of pathological objects (E). (TIF 4423 kb)

## Abbreviations

AD: Alzheimer’s disease
CBD: Corticobasal degeneration
CSF: Cerebrospinal fluid
FTD: Frontotemporal dementia
FTLD: Frontotemporal lobar degeneration
GFAP: Glial fibrillary acidic protein
HRP: Horseradish peroxidase
Iba-1: Ionized calcium-binding adapter molecule 1
IF: Immunofluorescence
IHC: Immunohistochemistry
LOAD: Late onset Alzheimer’s disease
MAP2: Microtubule-associated protein 2
MS: Multiple sclerosis
NFT: Neurofibrillary tangles
NT: Neuropil threads
NTB: Neurological Tissue Bank
PiD: Pick’s disease
PSP: Progressive supranuclear palsy
ROI: Regions of interest
TREM2: Triggering receptor expressed on myeloid cells 2
