# Supplementary figures and images for: YKL-40 (Chitinase 3-like I) is expressed in a subset of astrocytes in Alzheimer’s disease and other tauopathies
Source: J Neuroinflammation. 2017 Jun 9;14:118. doi: 10.1186/s12974-017-0893-7 (PMC5466718; doi:10.1186/s12974-017-0893-7)

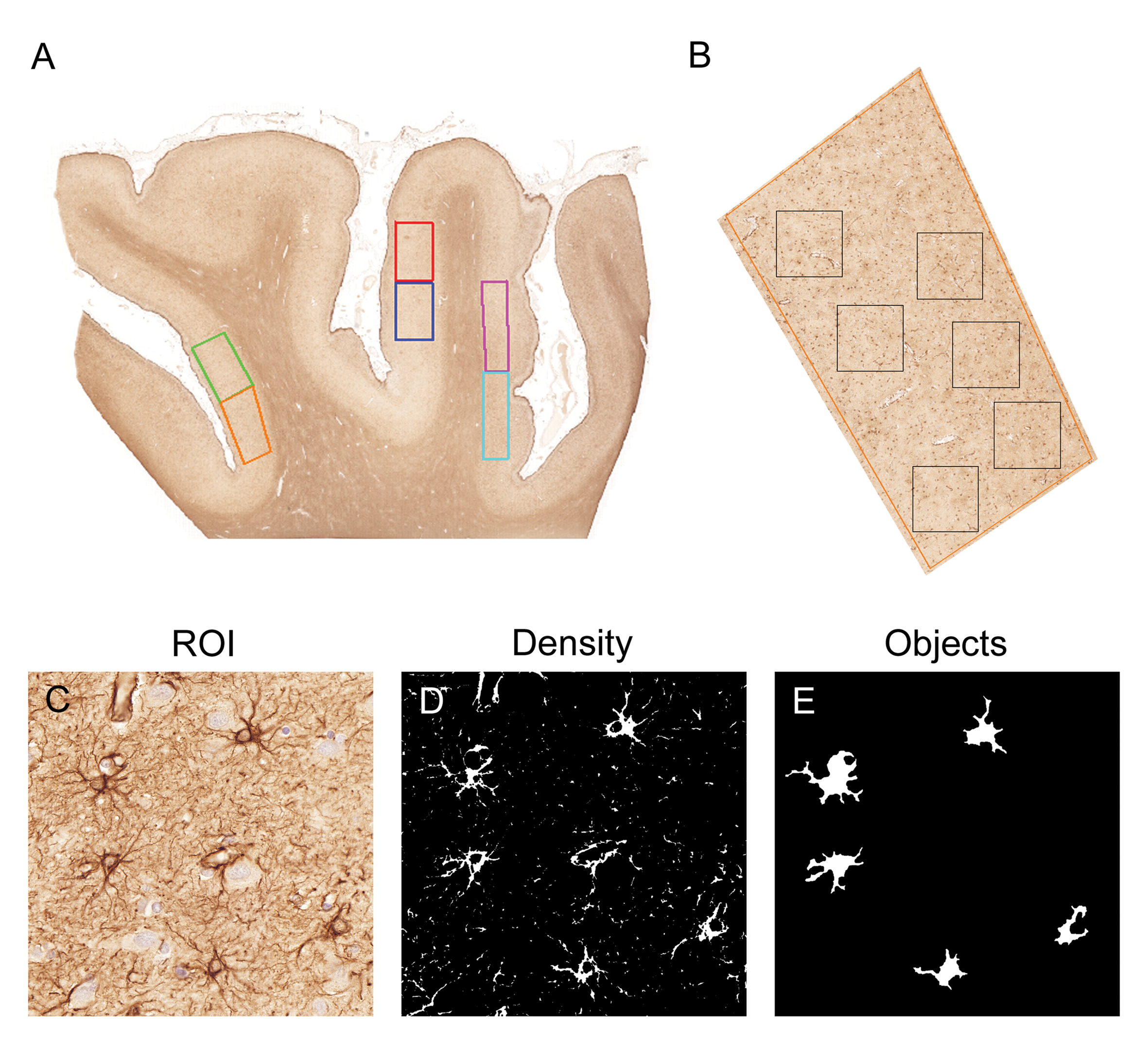

Supplement: Additional file 1: — Figure S1. Semi-automated method for pathological burden quantification. For all conditions tau and GFAP were assessed using a randomized computer-based quantification of patterns and severity in immunohistochemical stains. Cortical grey matter of each case was delimited blinded to clinical phenotypes (A). We developed an in-house algorithm that allows defining randomized regions of interest (ROIs) on a full-section scan (B–C), to compute density of protein expression (D) and to quantify the number of pathological objects (E). (TIF 4423 kb) [file 12974_2017_893_MOESM1_ESM.tif]
